# New products

**DOI:** 10.1155/S1463924687000129

**Published:** 1987


Journal of Automatic Chemistry/*Journal of Clinical Laboratory Automation*, Vol. 9, No. 1 (January–March 1987), pp. 51–56

### New products

### Robotic workstation

The Biomek 1000 is a new automated laboratory workstation from Beckman, well suited for operation in a variety of laboratory applications. Fast and accurate, this multi-task robotic workstation takes over tedious manual tasks and integrates the work of four different instruments. Biomek 1000 is a sample preparation system, a diluter/dispenser, a plate washer, and even a photometer. Its three-dimensional motion and interchangeable tools perform virtually any liquid handling operation in research and routine laboratories. Every step of complex methodologies can be performed at this single workstation—including photometry—which makes the Biomek 1000 ideal for immuno-assays such as ELISA, RIA, FIA, hybridoma screening and selection, and other bio-assays.

The Biomek 1000 performs much faster than a human operator. Plate-based enzyme immunoassays, for example, take 12 min from sample transfer to OD measurement, excluding incubations. A 10-fold, 96-tube serial dilution, followed by sample transfer to a 96-well plate with tip changes, can be completed in just over 3 min.

Biomek offers multiple pipetting options. Its single and eight tip pipette tools can be used to prewet tip, tip touch, blow out tip, change tip, mix, remove, and deliver sample at a specified well depth. A wide range of standard labware can be accommodated by the system. Biomek 1000 allows pipetting into multiwell plates, cryovials, microcentrifuge tubes and other tubes up to 13 × 100 mm size. Mix and match modular reservoirs are available, which can be user-customized to suit individual assay requirements.

Biomek 1000 can be easily programmed to perform even the most complex methodologies that involve liquid-handling operations. An IBM PC graphic controller with colour monitor displays the Biomek 1000 program in a menu-driven format which is simple enough for a computer novice.

The powerful software comprises an array-based assay language which allows the operator to program complex methodologies following individual laboratory techniques. Therefore Biomek is not limited to just a supplied program, since the user can dictate how the assay will be performed. The software guides the operator through each stage of the program with simple menus and prompts. Unlike conventional robots, Biomek 1000 does not need to be taught how to perform. Its special anti-collision feature automatically allows for different sizes of pipette tips, tubes, and plates when moving across the work surface.

*For further information contact Beckman Ltd, Progress Road, Sands Industrial Estate, High Wycombe, Buckinghamshire, UK. Tel.: 0494 41181.*

### Autosampler/fraction collector devices

To complement their 20·080 range of 80-position autosamplers, P S Analytical has recently introduced the 20·020 series. Based around the need to hold or collect larger volumes than normally available on commercial systems, the 20·020 series offers volumes of up to 100 ml. In the standard configuration it provides 20 60 ml containers. It will either operate in a stand-alone manner, or it can be used to control other devices, or it can be controlled by computer systems through a simple, but flexible, TTL logic interface. Using a patented washpot configuration it offers minimal carry-over and all turntable positions can be filled with samples or standards—there is no need to separate each real sample with a blank or wash solution. A sample bottle detector is included as standard so that only where sample bottles are present will the probe be lowered. At the end of a run the turntable can be reactivated by a single contact closure to reanalyse the samples. A signal is available, as the probe enters the sample, which can be used to trigger analytical integration or to set up another sequence of operations—for example activate pump to pull sample through an HPLC valve and subsequently trigger injection.

As with the 20·080 series, a range of probes is available: oil analysis, for online dilution, or for collection/sampling applications. A waterbath to hold samples at set temperature and a vial piercing mechanism are also offered.

The PSA 20·020 offers a number of features which make it an ideal system building block for automatic systems. Its simple but reliable design ensures that it operates continually without malfunction. OEM applications are also welcomed.

*For further details please contact P S Analytical, Arthur House, Cray Avenue, Orpington, Kent BR5 3TR, UK. Tel.: 0689 31632*

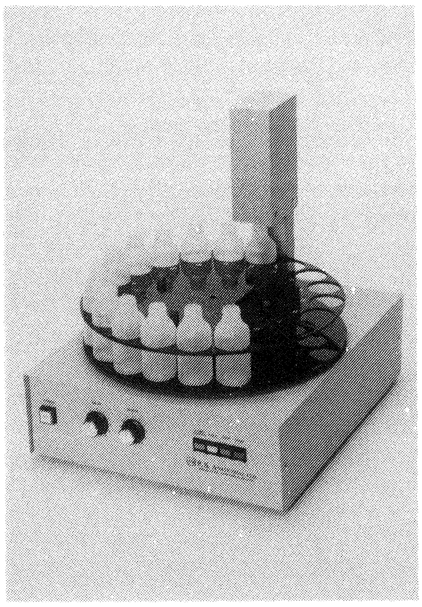

*An example of the 20·020 series of flexible large volume autosampler/fraction collection devices. The instruments are suitable for in-house system design and OEM applications.*

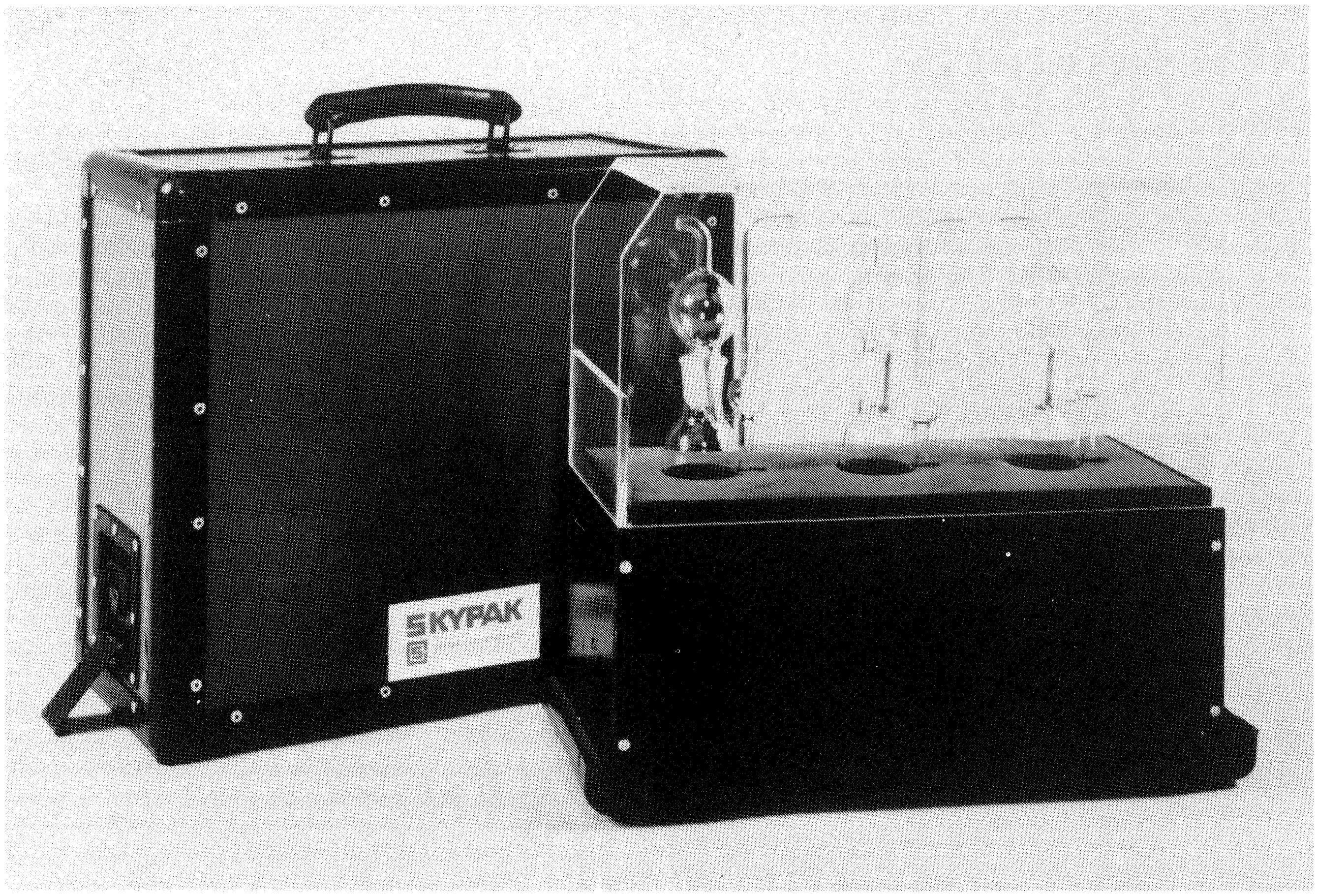

*Reusable containers to protect delicate medical and analytical equipment during transit are now available from Skypak Containers Ltd. They are designed not only to carry laboratory or field instruments, but also glassware and specimens, chemical, pharmaceutical or clinical.*

*The internal furnishings of each container consist of non-hygroscopic polyethylene foam, which provides secure cushioning, is resistant to most chemicals, including acids and alkalis, and is easy to clean and sterilize. The furnishings are precisely cut to the dimensions of the equipment, however unusual the shape or the number of pieces to be carried. They retain their strength at low cryogenic temperatures.*

*Full details are available from Skypak Containers Ltd, Unit 2, 500 Millbrook Road, Southampton, Hampshire, UK.*

### On-line analysis in remote locations

Guided Wave Inc. has combined process spectrophotometry, advanced fibre optics and data processing into a unique concept, which now being introduced on the World market by Guided Wave International in Sweden.

In order to monitor and control changes in a process the logical approach is to make observations directly in the dynamic environment, where the reaction takes place. This is true for laboratory and pilot plants as well as in the production process itself. However, process control instruments available today for *in situ* measurements suffer from limited performance and sensitivity apart from being expensive to maintain. Extracting a sample from the process via manual sampling equipment or automated sample transport systems creates other problems, including lag in the measurement and a need for maintenance and cleaning of the system on a regular basis.

The Guided Wave innovation offers new measurement possibilities and eliminates many of the problems with todays instrumentation. The solution is a system for remote composition analysis directly in the process—*in situ*—via fibre-optic spectrophotometry.

An optical fibre (waveguide) transmits light to the sensor-probe located inside the medium to be monitored. A second waveguide returns the absorption modified light to the instrument, where a spectra, containing information about the chemical composition of the medium, is generated. Instrument control and real time display of the results are provided through an or IBM PC equivalent computer. The spectral regions covered are both UV, VIS and NIR (from 230 nm up to 2200 nm).

*Distances* between the analyser and the point of measurement can be from 2 up to 500 meters, depending on the application and the spectral region of interest.

*For further information contact Guided Wave International AB Box 1264, S-251 12 Helsingborg, Sweden. Tel.: 46 42 11 14 60.*

### Plant automation

Ionics can assist plant chemists and process engineers in considering aspects of automating analytical techniques within their overall process and in the analysis of plant effluent. Ionics are currently working with major companies in the petrochemical waste-water control and electro-plating industries. The Digichem process wet chemical analyser has been used for many years to perform on-line analysis of both aqueous and organic solutions. Using various detectors and reagents, it can perform titrametric, colorimetric and ion selective measurements. Calculations are in engineering units to suit the application.

Such systems enable the process manager to base his decisions on a regular round-the-clock assessment of the plant's performance. Thus they can relieve operators of potentially hazardous manual analyses on hot or corrosive samples and increase the accuracy and repeatability of analyses.

*Further details are available from Ionics, 10 Statham Avenue, Lymm, Cheshire WA13 9NH, UK.*

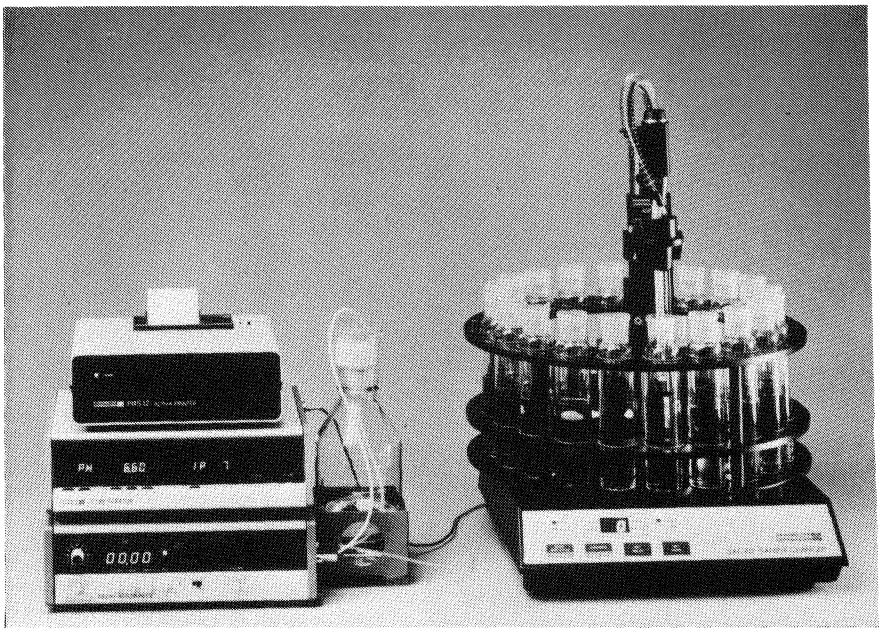

*For simplified determination of Chemical Oxygen Demand, Radiometer has introduced the DTS895 COD Analyser System, which, unattended, titrates up to 20 samples in the narrow digestion tubes. Transfer of sample from tube to titration beaker is thus eliminated, saving time and increasing analysis reliability. The system is modular, and comprises a titrator, an automatic burette, a printer and a sample changer. The sample changer has a removable turntable, making sample collection easy. The measuring electrode is a combined platinum/mercurous sulphate electrode.*

*The system calculates and prints out the analysis results in mg/l COD. Other units are user-programmable. Common digestion tubes, such as Strohlein, Behr, Merck, Tecator and Gerhardt, can be used with the system. Details from V. A. Howe & Co. Ltd, 12–14 St. Ann's Crescent, London SW18 2LS.*

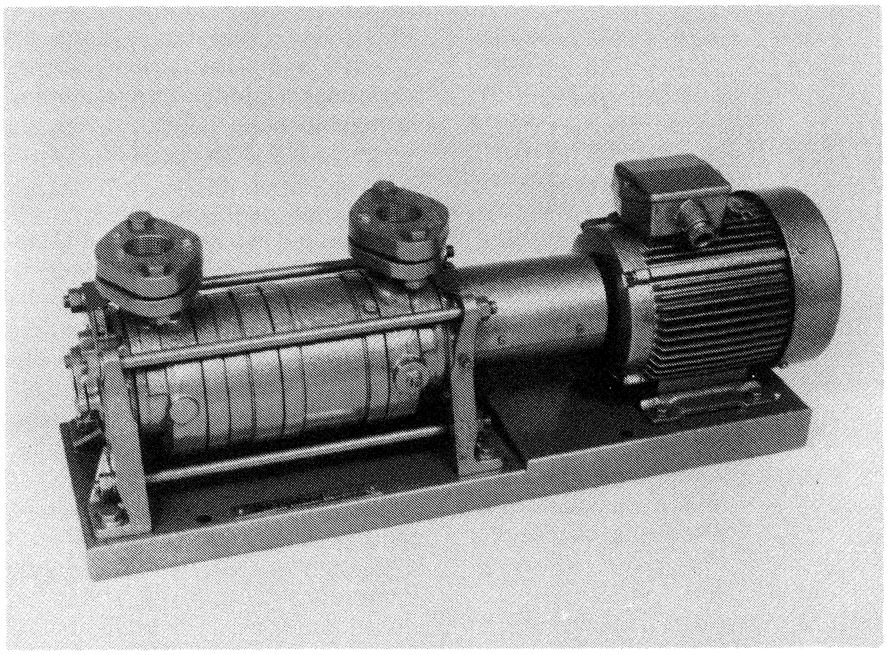

*The LOHE 25007 pump, a new, long coupled vacuum pump claimed to be more compact than traditional and monobloc pump designs, and to offer a competitive alternative to monoblocs in many applications. Available from SIHI-Ryaland Pumps Ltd, Bridgewater Road, Broadheath, Altrincham, Cheshire WA14 1NB, UK. Tel.: 061 928 6371.*

### Biotechnology back-up

Labomatic, designed to undertake high performance, medium pressure liquid chromatography is ideal for technicians seeking to purify chemicals up to 100 g. The manufacturer believes that this low-cost LC system will be of special interest to those involved in biotechnology. It is an important addition to Severn's product range because it provides a completely 'inert' system for purification of biologically active molecules. The package includes a gradient former, medium pressure piston pumps, pulse dampener, peak detectors, spectrophotometer, differential refractometer, fraction collectors and control instruments MPGC, FCPC and PGC solumns, especially suitable for silica packings, are also provided.

*For further information on the Labomatic, contact Steve Mitchell at Severn Analytical, 36 Brunswick Road, Gloucester GL1 1JJ, UK. Tel.: 0452 20306.*

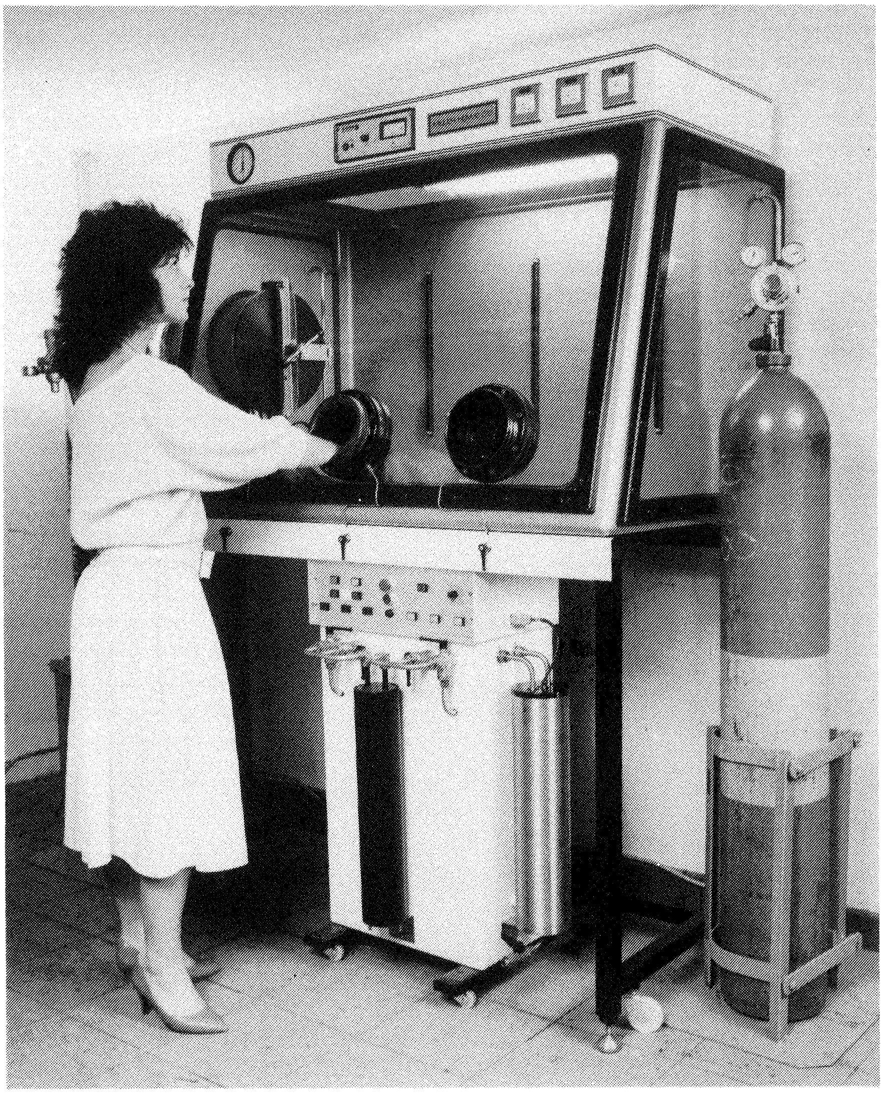

*A new, low-cost trace and percentage oxygen analyser manufactured by Systech Instruments Ltd is being specified by Miller-Howe to monitor gas purity in their glove boxes, and in addition they are also using it in the testing and commissioning of their eqiupment. The measuring principle is the electrochemical reduction of $O_2$ to hydroxide; the oxygen diffuses to a lead cathode where the following reaction takes place: $2Pb+O_2+2H_2O \rightarrow 2Pb(OH)_2$. The absence of bulk liquid electrolyte in the cell makes the system conveniently portable. The instrument weighs 3 kg, and can be mains or battery operated. It can be checked for accuracy by using ambient air as the calibration medium, and it is insensitive to flow rate changes through the analyser. Apart from its use to monitor chemical glove-boxes, the EC90 will have applications in cryogenics, microbiology, biotechnology, metallurgy, gas purity certification and semiconductor work. More information from Systech Instruments at Goodsons Industrial Mews, Wellington Street, Thame, Oxon OX9 3BX, UK. Tel.: 084421 6838.*

### Electrophoresis

Ultra-Violet Products Ltd (UVP) of Cambridge has announced the UK's first complete electrophoresis system for DNA/RNA analysis to sell for less than £1000. The Electro-4 includes a transilluminator, gel tank, power supply and camera which would normally cost more than £1500 when bought separately. The system will be able to carry out electrophoresis, visualization and photodocumentation at one station using two square feet of laboratory space.

UVP is expecting initial demand to come mainly from the education market, including universities, polytechnics, schools and research institutes. The system is small enough to allow students to participate in demonstations rather than just watching.

*Details from Ultra-Violet Products, Science Park, Milton Road, Cambridge CB4 4FH, UK. Tel.: 0223 355722.*

*Quaternary LC systems*

The PU4100 series from Philips Analytical ranges from a simple isocratic instrument to a fully automated quaternary chromatograph. Full upgradeability features in all machines. The isocratic system can be rapidly and inexpensively upgraded with plug-in modules, including binary and quaternary solvent accessories, column ovens, automatic injectors and full detector scanning.

The PU4100 Series has been designed for optimal performance whatever the separation requirement. Flow rates from 1 µl/min to 5 ml/min and a microbore autoinjector allow full use of low dispersion techniques, ideal in forensic and biotechnology environments.

Models in the range may be ordered as one of nine factory configured units. Alternatively, Philips offer the choice of individually selected options to build a laboratory-tailored system. At the top of the range, the PU4100/44 Research Quaternary LC offers sample sequencing, highest performance gradient programming, a highly stable column oven and scanning UV/VIS detection.

With true dual beam optics, the stability and low noise of the PU4110 UV/VIS detector are unrivalled. A wide wavelength range (190–700 nm) and a unique scanning option give exceptional versatility. This option rapidly and automatically scans LC peaks in high resolution or survey modes, and the eight spectrum memory store can be automatically dumped post-run to a printer/plotter. Capabilities for resolving overlapping peaks are offered by the facility for monitoring first or second derivative signals.

Both chromatograph and dectector are controlled from an intuitive user interface. With 32-character alphanumeric displays, the prompts are clear and the replies straightforward. To increase simplicity still further, method storage is standard.

*Further information from Pye Unicam Ltd, York Street, Cambridge CB1 2PX, UK. Tel.: 0223 358866.*

### Dual camera capability

Spectron Engineering has introduced an upgrade to their industry leading SE590 field-portable, data logging spectroradiometer. This portable spectral scanner which is used for remote sensing of agricultural, forestry and geological environments, now has the added ability to automatically switch between two spectral cameras.

The automatic camera switching option will allow near simultaneous acquisition of spectral data from two different spectral range cameras or two identical cameras. As an example, one camera would act as a reference and allow for verification of spectral data, such as with solar variations in field work. This option would allow a researcher to take a spectral scan of the 200 nm–1100 nm range with as little as a 1.37 nm dispersion per detector element.

This new addition incorporates their existing spectral cameras already in the field which have the ability to simultaneously acquire data in 256 bands in as little as 1/60 s. The cameras scan in the range of 200 nm through 2.5 μm with the ability of the unit to direct the output either to the internal tape drive or to an optional RS232C communications port for external data manipulation.

Once acquired, a spectrum can be displayed on an oscilloscope via the scope output; the amplitude at each wavelength can be displayed on the built-in LED display; or it can be recorded onto the tape. With options, a ratio may be taken of the current spectrum to a previously acquired one (such as a reference spectrum of barium sulphate) and the spectrum or spectral ratio can be printed or plotted. Direct referenced computation of spectroflectance ratios give power unusual even in most laboratory spectrometers.

Printer, X/Y plotter, ratio computation and other computing options expand the self-contained system capabilities to provide hard-copy processed data. The system operates a printer to produce a tabular printout of spectral or spectral ratio data; it can also print a graphic representation or produce a spectral plot on a x/y recorder.

The controller includes an RS-232C output to transmit spectra (with ID codes) from tape to a remote computer system for building the desired data base for analysis. Analysis software is currently available for several microcomputers and commercial data reduction services are also expected to be available.

In addition to providing continuous spectral data, the system is also compact and simple to use, ideal for field use. Built-in optics allow simply aiming the spectral head at the area of interest. The spectrum acquired can be reviewed in the field on the internal LED display or by coupling a portable oscilloscope. The spectrum can be recorded with a single keystroke. The unit operates on internal rechargeable batteries (a battery charger is supplied). For extended field use, it can be plugged into a 12 V to 15 V dc external source.

A synchronization pulse is provided to trigger a photographic camera to record the scanned area, an important feature in aerial work. An auxilary lens adapter with a 1 degree spot or 15 degree wide angle lens, a fibre optic probe, and multiple signal averaging capabilities are some of the available options.

The standard head continuously covers 370–730 nm and custom bands can be specified as well. It is even possible to add discrete IR detectors; for example, to cover the middle infra-red bands of the Thematic Mapper (Landsat satellite program) out to 2.5 micron.

*The compact, portable SE590 spectroradiometer system has a base price of $9500; delivery is typically six weeks. For information contact: Spectron Engineering, Inc., 800 W. 9th Avenue, Denver, Colorado 80204, USA. Tel.: 303 623 8987.*

### Sample preparation

A 150-page book, just published by Analytichem International, contains copies of papers presented at Analytichem's symposium in Philadelphia in 1985. Comprehensively illustrated, they demonstrate the use of the technology in biomedical, environmental and industrial applications.

Specific topics covered in the proceedings volume include the rationale of method development; rapid separation of lipid classes in high yield and purity; preparation of urine for analysis of oestrogen conjugates; sample matrix considerations in method development; selective isolation of PAH and PCB at extremely low levels in water samples; and the sampling of priority pollutants in wastewaters.

*Copies from Analytichem International, PO Box 234, Cambridge CB2 1PE, UK. Tel.: 0223 328177.*

### Enhanced UV-260

In response to customers' demand for additional spectral storage capability, Shimadzu have released an enhanced version of the UV-260 Double Beam spectrophotometer. The new instrument is available in two formats: UV-265FS, single floppy disk version, and UV-265FW, dual floppy disk version.

UV-265 resembles the UV-260 in appearance, having a large sample compartment, accessible from three sides, a VDU which operates independently to recorder plus a thermographic printer/plotter. The UV-265 version has a disk drive inserted between the instrument main body and the printer/plotter.

The instrument incorporates all the features of the UV-260, to achieve a variety of data processing functions, such as arithmetical calculations between spectra, measurement of derivative spectra, multi wavelength measurement, calculation of spectra areas, recording of change in photometric values against time, detection of peak wavelength, wavelength programming, plus high level data processing via the User Programmable Command Chain Mode.

The addition of the dual sided, double density (1.2 M byte) floppy disk drives, allows for as many as 75 spectra (3500 data points per spectrum), together with measuring conditions, time, date, plus comments to be stored and recalled at any time for comparison with spectra obtained later. Calculation curves, time course spectra, command chain programs, plus instrument measuring conditions, can also be stored, independently on a dedicated disk or collectively, together with other formats on a multi-function disk.

*Further details from V. A. Howe Co. Ltd, 12–14 St. Ann's Crescent, London SW18 2LS. Tel.: 01 874 0422.*

### XTRA random access

*TRAF protected carry-over free operation*

The Technicon RA-XT system benefits from the technology of the RA-1000 line of instruments by using an immiscible oil – TRAF – to protect both sample and reagent probes from carry-over allowing assays to be run in true random access mode, sample by sample.

*26 chemistries on line*

From a menu of 128 chemistries held in memory, RA-XT will access from 26 chemistries at any one time, including sodium, potassium and bicarbonate by indirect ISE. Although this covers 95% of the commonly requested tests, change-over to another configuration of methods takes less than 1 min.

*Refrigeration of 40 ml reagent boats*

Reagents can be held on-system in volumes up to 40 ml. The reagent area can be kept at 4 °C to 8 °C with the use of an optional cooling unit, allowing full use of the large reagent boats in the area of lower work-load tests.

*Touch-sensitive screen, menu-driven software*

Removing the need to commit codes to memory, the touch-sensitive screen with its menu-driven software leads the user through the operation of the system, allowing ease of use and rapid training.

*Intelligent random access*

As an alternative to carrying out tests in user-defined order, the intelligent random access mode schedules longer-incubation tests ahead of others to maximize throughput. The system maintains a sample-by-sample regime to enhance sample to report times and avoid the delays typical of the batch approach.

*Multiple sample-container facilities*

The sample handling system is designed to use a variety of containers including original samples and micro-tubes in addition to traditional sample cups. These can be used together in the same analytical run.

*Floor-standing module*

The Technicon RA-XT system can be delivered as a floor-standing unit on its own console, especially useful when the refrigeration unit is specified, or when bench space is at a premium.

*Data management and bar code sample identification*

Security of sample identification and release from the need to work-list are the benefits of the bar code reader and data management options on RA-XT. Additionally validation of results, work-load management, extensive quality control regimes, patient detail storage, customized print-outs and a variety of reporting options including cumulative are amongst the features of the system. CAP work-load statistics are also available on a daily and cumulative basis.

*Compatible methods*

Over 90 documented methods, from phosphate to fructosamine and from theophylline to throxine have been developed for the RA-1000/RA-500 systems in addition to many more not formally reported. The new RA-XT system is fully compatible with this extensive library of methods, offering the flexibility needed to service all areas of the laboratory work-load.

*Details from Francis Hooley, Technicon Instruments Company Ltd, Evans House, Hamilton Close, Houndmills, Basingstoke, Hampshire RG21 2YE, UK. Tel.: 0256 29181.*

### Emission spectrometers

Designed for elemental analysis of irons, steels, aluminium alloys and other non-ferrous materials, the PV8030 Series' rugged construction, vibration-proof mountings and efficient temperature control ensure stable operation in the severest of industrial environments.

With full 1 m optics covering a wavelength range of 177–410 nm, the instruments offer higher resolution than others in their class. Either a 50 Hz or 50–500 Hz programmable monoalternance source unit can be fitted, the variable frequency unit allowing determinations to be completed in as little as 4 s.

Variable burn conditions may be programmed during the course of a single analysis. With a 500 Hz source, this permits simultaneous optimization of element detection limits and accuracy of major constituent determinations.

Two factory calibration packages are available – one for users whose requirements are confined to steels alone, while the other permits the analysis of both irons and steels. The remaining capacity may be filled with additional elements to match the majority of manufacturers' requirements.

Spectrometer functions are controlled and monitored by built-in microprocessor electronics operating in conjunction with an IBM compatible Philips P3100 personal computer. The software uses menus and dialogues to guide the operator through every stage. Complete measuring routines can be stored and then recalled by a single command, so shop-floor personnel can easily handle all daily procedures.

*Details from Sales Promotion Department, Enquiry Handling Section, Pye Unicam Ltd, York Street, Cambridge CB1 2PX, UK.*

